# Supplementary figures and images for: Exploring the Molecular Mechanism and Role of Glutathione S-Transferase P in Prostate Cancer
Source: Biomedicines. 2025 Apr 26;13(5):1051. doi: 10.3390/biomedicines13051051 (PMC12109251; doi:10.3390/biomedicines13051051)

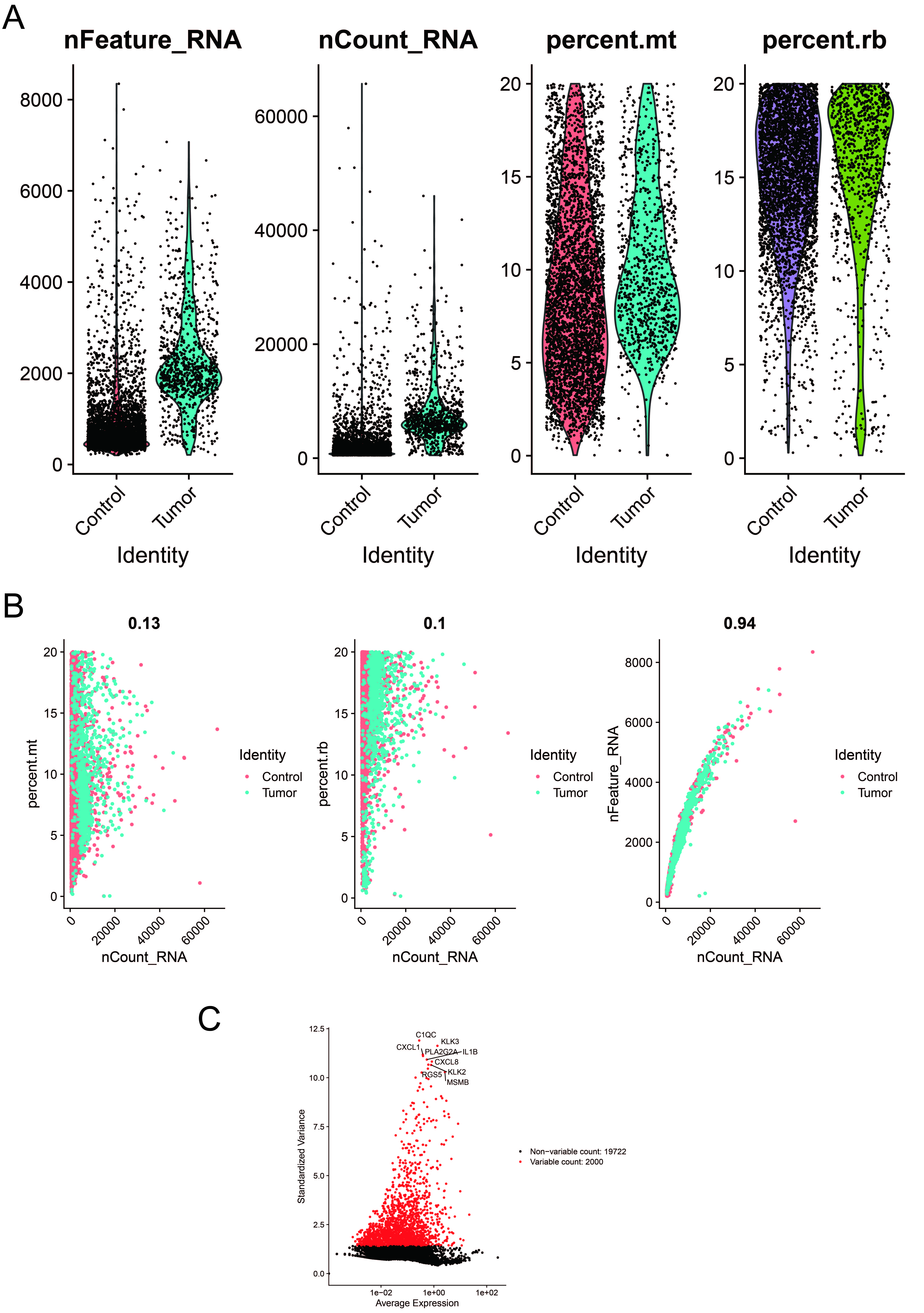

Supplement: Supplementary file 1 [file biomedicines-13-01051-s001.zip › Supplemental Figure S1.jpg]

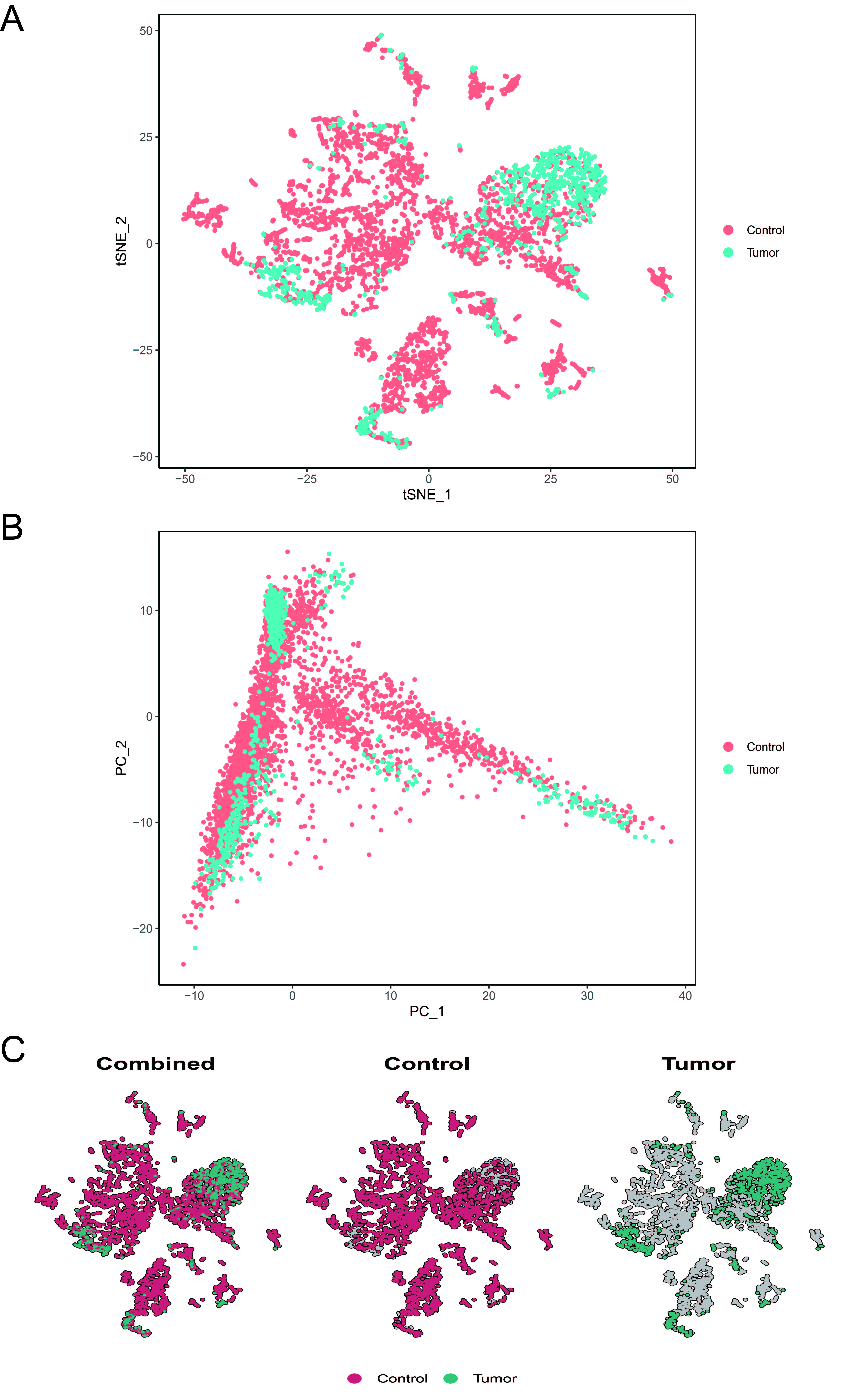

Supplement: Supplementary file 1 [file biomedicines-13-01051-s001.zip › Supplemental Figure S2.jpg]

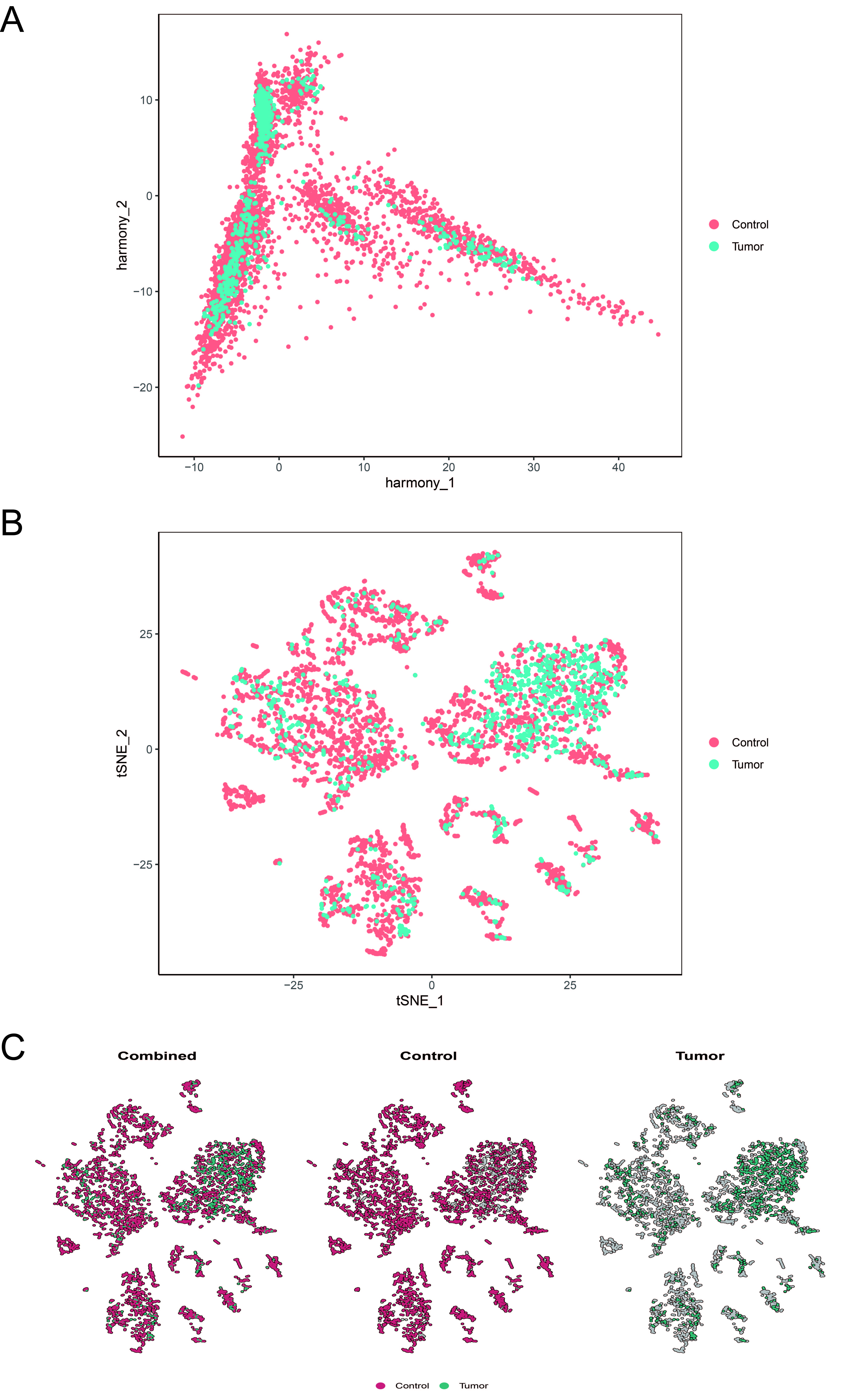

Supplement: Supplementary file 1 [file biomedicines-13-01051-s001.zip › Supplemental Figure S3.jpg]
